# Case Report: Third eyelid flap for correction of proptosis-induced lateral strabismus in two dogs

**DOI:** 10.3389/fvets.2025.1602473

**Published:** 2025-09-12

**Authors:** Byung-Ju Jeon, Joon-Young Kim

**Affiliations:** Department of Veterinary Ophthalmology, College of Veterinary Medicine, Konkuk University, Seoul, Republic of Korea

**Keywords:** lateral strabismus, third eyelid flap, proptosis, ocular surgery, dogs

## Abstract

This case report describes the use of a third eyelid flap to correct proptosis-induced lateral strabismus in two dogs: a 3-year-old castrated male Pomeranian (Case 1) and a 2-year-old intact female Bichon Frise (Case 2). In both cases, the third eyelid was sutured to the dorsolateral conjunctiva using a horizontal mattress suture. However, conjunctival elongation at the suture site compromised flap function, necessitating reoperation. In Case 2, a second horizontal mattress suture was added to improve stability. Following reoperation, Case 1 achieved complete correction of lateral strabismus by day 42, with transient third eyelid protrusion and corneal thinning resolving within two weeks. In Case 2, mild residual strabismus remained, but cosmetic improvement was substantial, and third eyelid inflammation resolved similarly. These cases demonstrate the potential utility of a third eyelid flap as an adjunctive method for correcting lateral strabismus secondary to proptosis in dogs, offering a less invasive alternative to conventional incisional surgery.

## 1 Introduction

Ocular proptosis is an ophthalmic emergency characterized by the partial or complete displacement of the globe beyond the orbit, typically caused by a sudden forward shift that traps the eyelids behind the equator of the globe (1).

Proptosis can result in severe ocular and periocular damage, often necessitating enucleation or globe replacement surgery, depending on the severity of the injury. Even with timely and appropriate intervention to preserve the globe, long-term complications such as blindness, strabismus, mild exophthalmos, lagophthalmos, corneal sensory deficits, keratoconjunctivitis sicca, exposure keratitis, glaucoma, and phthisis bulbi may still develop. Among these complications, strabismus arises from damage to the extraocular muscles, with lateral strabismus being the most frequently observed. This condition typically results from damage to the medial rectus muscle, which is the shortest and most vulnerable of the extraocular muscles, as noted in Gelatt's *Veterinary Ophthalmology* (2) and other textbooks (3–5). A study by Ali et al. (6) investigating traumatic proptosis and its postoperative complications in small-breed dogs reported that all 15 dogs exhibited medial rectus muscle damage following globe replacement surgery.

Strabismus is important not only for aesthetic reasons but also for its functional implications. It causes ocular misalignment, leading to diplopia, as each eye projects different images onto the brain. In young individuals, the brain compensates by suppressing input from one eye, which can result in amblyopia, leading to reduced visual acuity and binocular vision abnormalities. However, the effects of diplopia and amblyopia on vision in veterinary patients have not been studied (3, 7). Treatment options for strabismus in humans include incisional surgery of the extraocular muscles and chemotherapy with botulinum toxin A (7). Although similar surgical techniques are commonly used in veterinary practice, research on their efficacy in dogs is limited.

Given the limitations of conventional methods, this case report explores a third eyelid flap as a viable, less invasive adjunct to surgical correction following proptosis.

## 2 Case description

### 2.1 Case 1

**Figure 1:**
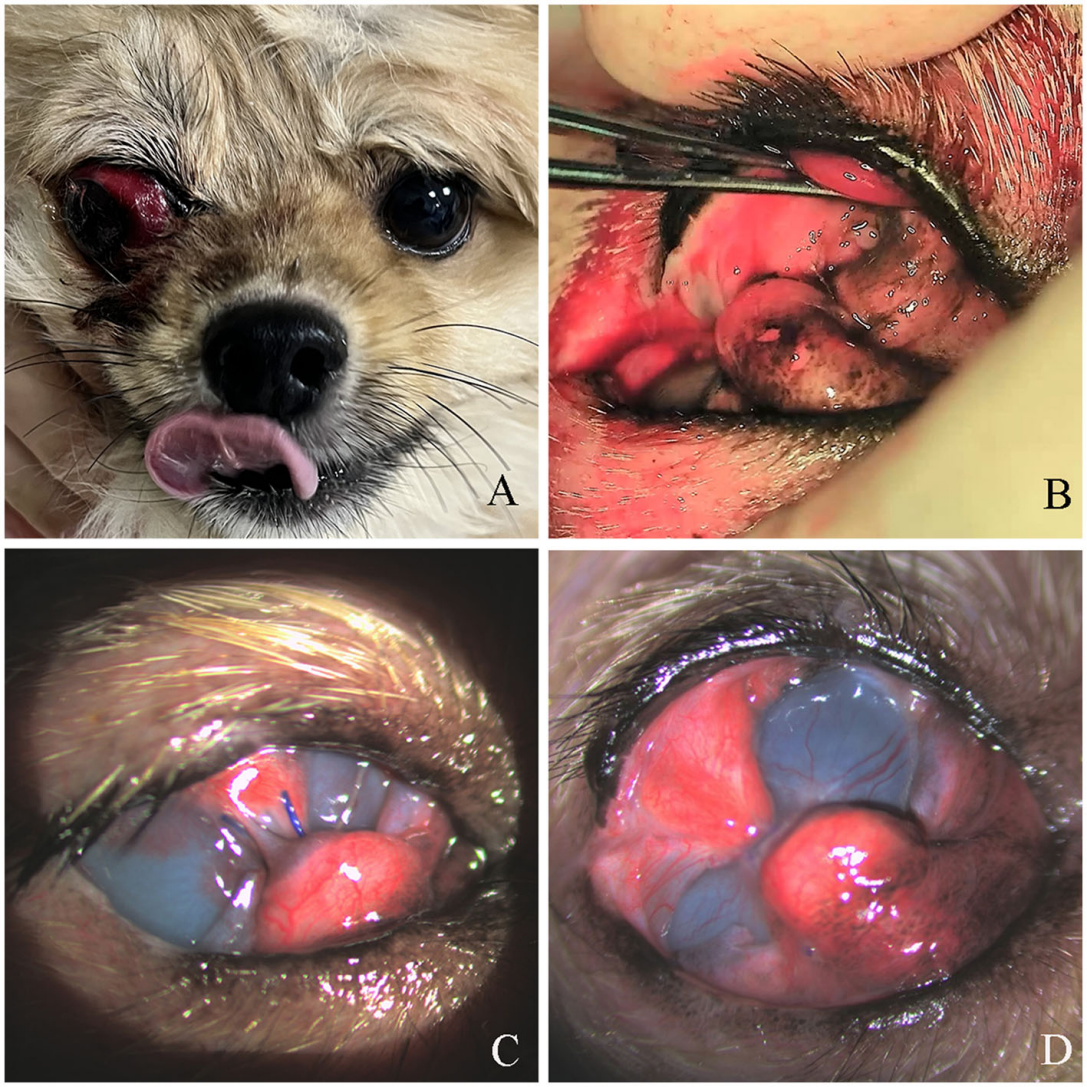
Preoperative appearance and third eyelid flap progression in Case 1. **(A)** Proptosis of the right eye with lateral strabismus following blunt trauma. **(B)** Immediate postoperative appearance after third eyelid flap. **(C)** Conjunctival elongation at the suture site 17 days postoperatively. **(D)** Recurrent conjunctival elongation without suture exposure, 14 days after reoperation.

A 3-year-old castrated male Pomeranian was presented to the Konkuk Veterinary Medical Teaching Hospital (VMTH) with proptosis of the right eye following blunt ocular trauma. General ophthalmic examination revealed vision loss, corneal ulceration, intraocular hemorrhage, and lateral strabismus (Figure 1A). To prevent further damage, globe replacement and temporary tarsorrhaphy under local anesthesia were performed one day before definitive surgery.

The surgical procedure was conducted in two stages under general anesthesia. In the initial stage, evisceration with intraocular silicone prosthesis was performed using standard techniques (8) to manage severe intraocular damage from blunt trauma. Subsequently, a third eyelid flap was used to correct lateral strabismus. After confirming the integrity of the third eyelid, it was sutured to the dorsolateral conjunctiva using 5-0 polyglactin (Vicryl; Ethicon, Somerville, NJ, USA) in a horizontal mattress pattern. During suturing, anchoring was achieved on the T-shaped cartilage of the third eyelid to maintain conjunctival integrity and prevent tearing (Figure 1B). Upon verifying adequate eyeball support from the third eyelid, temporary tarsorrhaphy was performed for stabilization using 5-0 nylon (Dafilon; B. Braun, Melsungen, Germany). Oral cephalexin (30 mg/kg; Kukje, Gyeonggi, Korea), carprofen (2.2 mg/kg; Zoetis, NJ, USA) q12h, and topical ofloxacin (Tarivid; Santen, Osaka, Japan) q12h were prescribed. Throughout the three-day hospitalization, no notable clinical abnormalities were observed except for mild ocular discharge.

**Figure 2:**
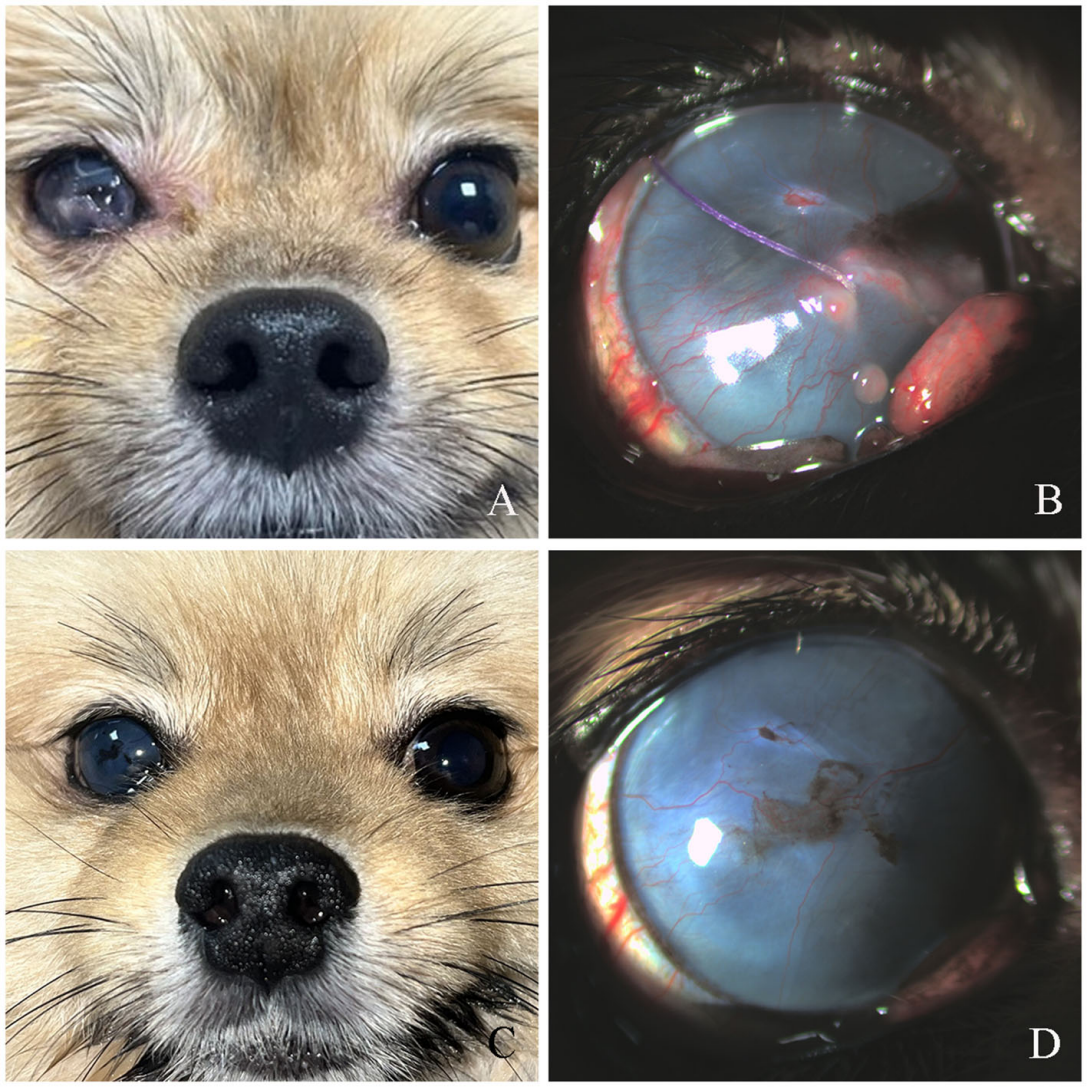
Post-flap removal and long-term outcomes in Case 1. **(A)** Complete correction of lateral strabismus, 42 days after reoperation. **(B)** Residual conjunctival tissue, third eyelid protrusion, and focal keratitis with corneal thinning were observed. **(C, D)** At 300 days post-reoperation: no recurrence of strabismus.

Seventeen days postoperatively, the temporary tarsorrhaphy was removed for evaluation. Conjunctival elongation at the suture site was observed at that time, which resulted in failure of the third eyelid flap to achieve its intended function (Figure 1C). To correct strabismus and prevent corneal complications, reoperation was performed using the previous method, excluding tarsorrhaphy. Postoperative care included topical neomycin-polymyxin B sulfate-dexamethasone (Maxitrol; Novartis, Basel, Switzerland) q12h and 0.2% cyclosporine ointment (Optimmune; MSD, NJ, USA) q12h. Fourteen days after reoperation, recurrent conjunctival elongation was observed (Figure 1D). However, because the suture was not exposed, the risk of corneal damage was considered low, and no further reoperation was performed. At 42 days post-reoperation, complete correction of lateral strabismus was confirmed after suture removal (Figure 2A). Concurrent findings included keratitis with residual conjunctival tissue, moderate corneal neovascularization, and slight protrusion of the third eyelid (Figure 2B). Notably, the cornea in contact with the suture was thinned and fluorescein staining was negative. Fifty-three days post-reoperation, redness and swelling of the third eyelid, corneal neovascularization, and residual conjunctival tissue showed improvement, although corneal pigmentation persisted. At 300 days post-reoperation, corneal fibrosis, neovascularization, and pigmentation were evident, with no recurrence of lateral strabismus (Figures 2C, D).

### 2.2 Case 2

**Figure 3:**
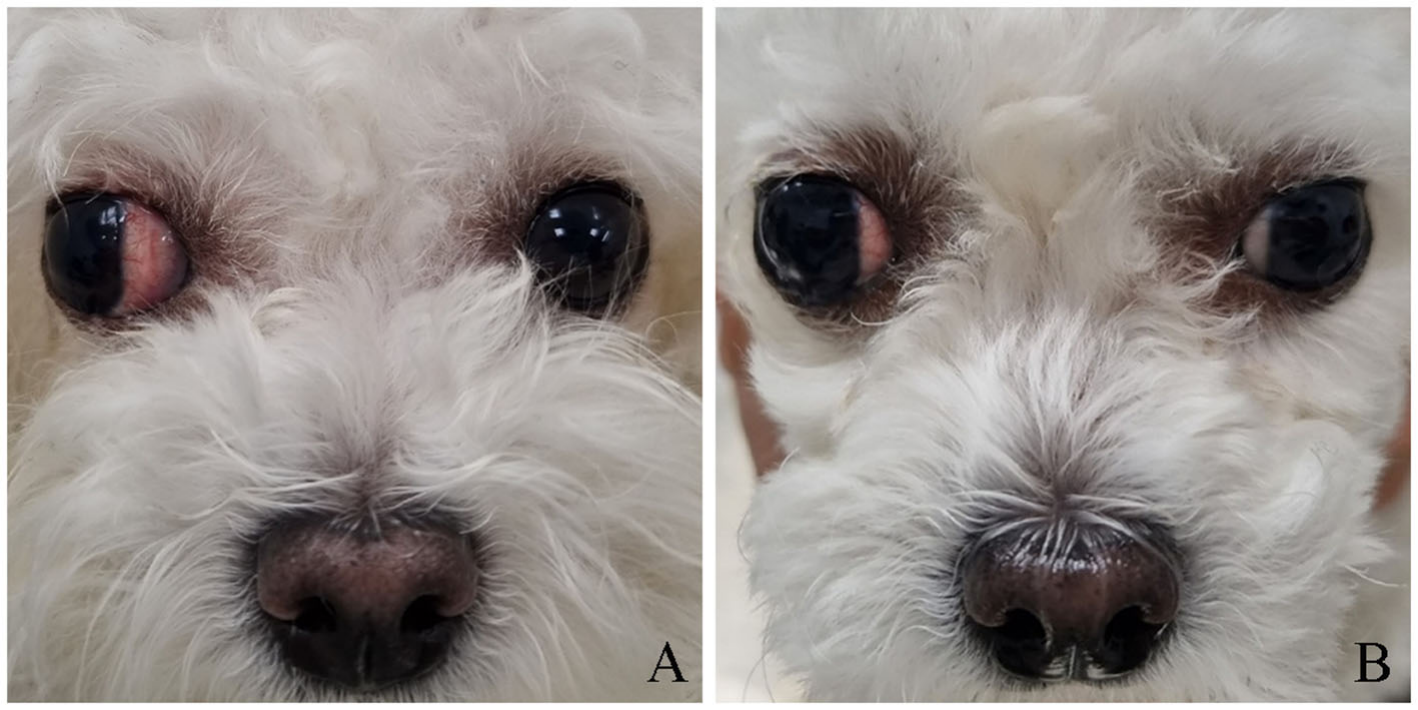
Preoperative and postoperative appearance in Case 2. **(A)** Lateral strabismus secondary to traumatic proptosis. **(B)** At 38 days post-reoperation.

A 2-year-old intact female Bichon Frise underwent globe replacement and temporary tarsorrhaphy at another local animal hospital following proptosis of the right eye caused by blunt trauma. Two weeks later, the dog was referred to the Konkuk VMTH with lateral strabismus. General ophthalmic examination revealed no abnormalities except for conjunctival hyperemia and lateral strabismus (Figure 3A). Correction of lateral strabismus was performed on the same day. The surgical approach, except for evisceration, was consistent with that used in Case 1. Oral cephalexin (30 mg/kg), carprofen (2.2 mg/kg) q12h, and topical ofloxacin q12h were prescribed for postoperative care.

**Figure 4:**
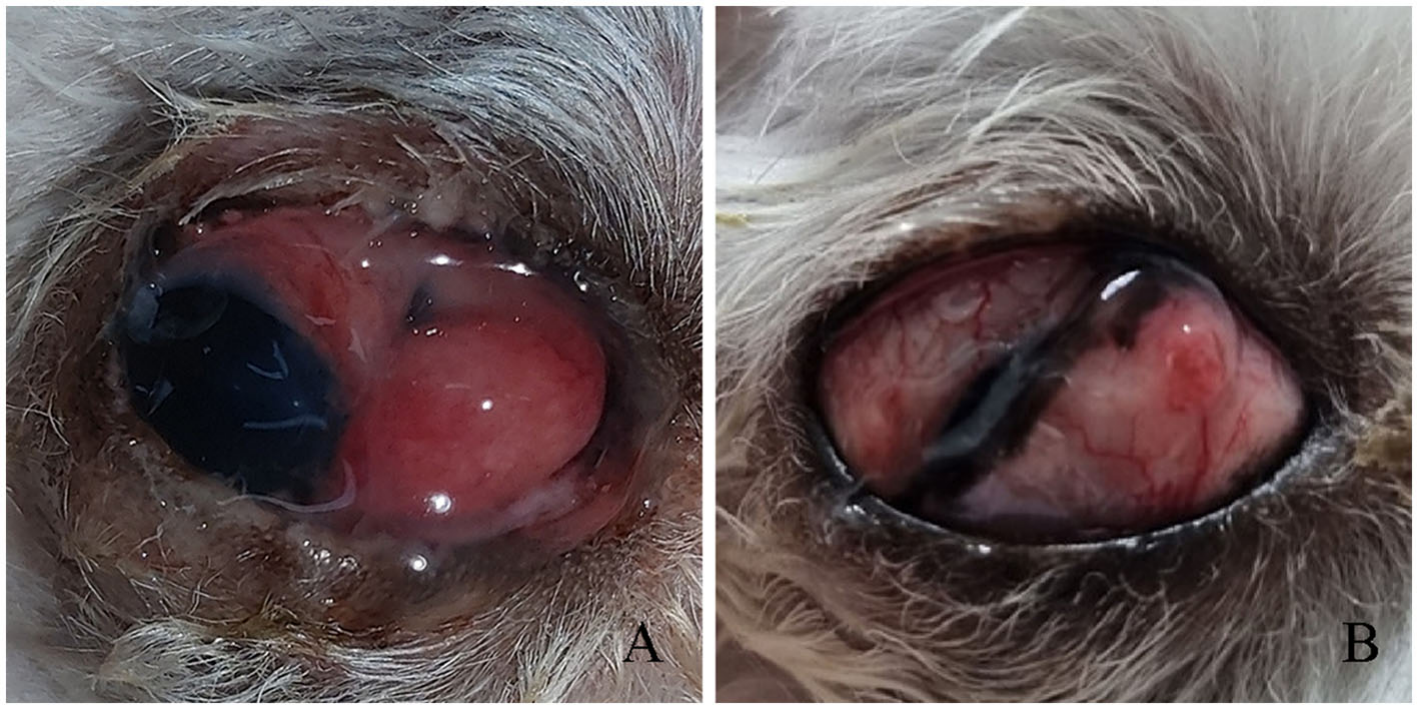
**Third** eyelid flap progression in Case 2. **(A)** Conjunctival elongation at the suture site, 14 days postoperatively. **(B)** At 21 days post-reoperation, the flap remained stable without recurrence.

Fourteen days postoperatively, the temporary tarsorrhaphy was removed for assessment. As in Case 1, conjunctival elongation at the suture site was observed, necessitating reoperation (Figure 4A). The procedure was similar to the previous one; however, in Case 2, an additional horizontal mattress suture was used to prevent recurrence and enhance stability. Topical ofloxacin q12h was prescribed for postoperative care. At 21 days post-reoperation, the third eyelid flap remained stable, and the topical eye drops were switched from ofloxacin to neomycin-polymyxin B sulfate-dexamethasone q12h (Figure 4B). Sutures were removed 32 days after reoperation. The third eyelid showed slight protrusion and redness. By 38 days post-reoperation, the third eyelid had returned to its normal position (Figure 3B). Although minor residual lateral strabismus persisted, the overall appearance had significantly improved compared with that before surgery. Despite the lack of an extensive long-term follow-up in Case 2, a telephone interview 755 days post-reoperation confirmed no recurrence of lateral strabismus or other ocular abnormalities.

## 3 Discussion

Ocular proptosis is a major cause of lateral strabismus in dogs, typically resulting from damage to the medial rectus muscle, the shortest extraocular muscle (2). According to Ali et al. (6), medial rectus damage was confirmed in 15 dogs with ocular proptosis. Following globe replacement surgery, lateral exotropia and dorsolateral strabismus were observed in 20% and 26% of cases, respectively.

Correction of lateral strabismus is essential not only for aesthetic reasons but also for preventing functional impairment. In humans, such impairments manifest as diplopia, visual confusion, and abnormal head posture (7). Unlike vestibular-related conditions that may not require surgery, non-vestibular strabismus typically necessitates surgical intervention. Although strabismus surgery is well established in human medicine, its application in veterinary medicine remains less common. This disparity largely arises from a limited understanding of how strabismus affects canine vision, as the visual processing mechanisms in dogs differ from those in humans (3). In humans, strabismus is often treated with extraocular muscle incisional surgery or chemodenervation using botulinum toxin A (7). Although similar surgical methods are employed in veterinary practice, research on their efficacy in dogs remains limited.

Third eyelid flaps are primarily used to protect the corneas of patients with corneal ulceration. However, when the third eyelid is sutured to the dorsolateral conjunctiva, it exerts an inward pull on the eyeball, which has led to its use in correcting lateral strabismus. In both cases, the third eyelid flap successfully corrected lateral strabismus. In Case 1, complete correction was confirmed upon suture removal, and the improvement was maintained during a long-term follow-up 300 days postoperatively. In Case 2, although slight strabismus persisted at 38 days postoperatively, there was substantial improvement compared to the preoperative condition. Long-term follow-up was not possible because of the owner's circumstances; however, the owner reported no recurrence of lateral strabismus or other ocular abnormalities during a telephone consultation 755 days after reoperation. The duration of flap application was 59 days and 46 days in Cases 1 and 2, respectively. These findings support the potential utility of the third eyelid flap in correcting lateral strabismus secondary to proptosis. This technique may serve as an effective, less invasive alternative or adjunct to conventional extraocular muscle surgery in selected cases.

The mechanism by which the third eyelid flap aids in strabismus correction involves the mechanical force generated when the third eyelid is anchored to the dorsolateral conjunctiva. This suture placement applies sustained medial traction on the globe, counteracting the outward deviation caused by medial rectus muscle damage. By repositioning the globe centrally, this method offers a temporary but effective means of realigning the eye during the healing phase.

Conjunctival elongation was already evident at the time of temporary tarsorrhaphy removal in both cases, suggesting that flap failure may not have been solely attributable to premature removal. Instead, it is plausible that progressive conjunctival elongation developed postoperatively as a result of sustained mechanical stress and tension concentrated at the suture site. In both cases, reoperation was necessitated by conjunctival elongation, which not only compromised the stability of the third eyelid flap, reducing its pulling effect, but also posed a risk of corneal irritation from the sutures, potentially leading to corneal ulceration. In Case 1, recurrent conjunctival elongation was observed after reoperation. In Case 2, to prevent recurrence, two horizontal mattress sutures were applied, successfully preventing conjunctival elongation and suture exposure, resulting in a stable flap. These findings suggest that applying two horizontal mattress sutures may be a useful refinement in future procedures, as it improves flap stability and prevents conjunctival elongation by distributing tension more evenly.

Alterations in the third eyelid were observed in both cases. In Case 2, slight protrusion and redness were noted on the day the sutures were removed. In contrast, Case 1 exhibited more pronounced inflammation and structural deformation of the third eyelid, possibly because the flap was maintained for 13 days longer than in Case 2. Additionally, severe ocular damage resulting from the initial proptosis in Case 1 may have exacerbated these effects. Notably, inflammation in Case 1 resolved 11 days later, leaving only slight protrusions.

This report is limited by the absence of objective criteria for evaluating strabismus correction; all assessments were based on subjective clinical observation. Additionally, long-term assessment for Case 2 was not performed by clinical follow-up and relied solely on information obtained through a telephone interview with the owner. Most importantly, this report is a case study presenting only two instances.

In conclusion, these cases demonstrate the efficacy of third eyelid flaps in correcting lateral strabismus caused by proptosis. This method may serve as an additional treatment option in conjunction with conventional incisional surgical procedures. Future studies are recommended to investigate specific outcome measures, such as the relationship between suture number and conjunctival elongation, or between flap duration and strabismus correction success rate. These insights would help refine surgical protocols and assess the long-term efficacy of third eyelid flaps for lateral strabismus correction.

## Data Availability

The original contributions presented in the study are included in the article/supplementary material, further inquiries can be directed to the corresponding author.
